# Adaptation and field testing of an observational assessment tool for autism diagnosis in Western Kenya

**DOI:** 10.3389/fpsyt.2026.1840667

**Published:** 2026-08-18

**Authors:** Kristin Fauntleroy-Love, Chelagat Saina, Eren Oyungu, Amira Nafiseh, Anita Jerop Rutto, Regina Amondi, Megan S. McHenry, Rebecca McNally Keehn

**Affiliations:** 1 Department of Pediatrics, Emory University School of Medicine, Atlanta, GA, United States; 2 Moi University, Eldoret, Kenya; 3 Moi Teaching and Referral Hospital, Eldoret, Kenya; 4 Academic Model Providing Access to Health Care (AMPATH), Eldoret, Kenya; 5 Department of Pediatrics, University of California, San Francisco, San Francisco, CA, United States; 6 Department of Pediatrics, Indiana University School of Medicine, Indianapolis, IN, United States

**Keywords:** assessment tools, autism, cultural adaptation, diagnosis, LMIC

## Abstract

**Background:**

The development and implementation of culturally appropriate, easy-to-administer, open-source assessment tools are critical for advancing autism detection and research in low- and middle-income countries.

**Methods:**

The ASD-PEDS, an open-source semi-structured observational tool for assessing autism-related behaviors in young children, was adapted for use in Kenya via iterative refinement throughout the process of forward–backward translation (English–Kiswahili), cognitive interviews with clinicians and caregivers, and field testing to ensure cultural relevance.

**Results:**

Cognitive interviews revealed culturally normative early social communication and play skills aligned with key behaviors assessed by the ASD-PEDS. Field testing further identified targets for adaptation including development of bilingual administration guidelines and cultural modifications to assessment activities. During field testing, ASD-PEDS categorical risk matched the child’s diagnosis in 100% of cases, demonstrating initial alignment with clinical classification.

**Conclusions:**

With minor adaptations, the ASD-PEDS demonstrates preliminary feasibility as an observational assessment tool to support standardized autism evaluation of young children in low-resource settings. Given the early-stage nature of this adaptation study, findings should be interpreted as preliminary. Study of psychometric performance in a heterogeneous larger sample of children is a necessary next step for cultural validation.

## Introduction

Autism spectrum disorder (ASD), hereafter referred to as autism, is a complex neurodevelopmental disorder characterized by differences in social communication and play along with the presence of restricted and repetitive behaviors (1). Globally, autism affects approximately 1 in 100 individuals, with the average age of diagnosis around 60 months (2, 3). Ninety-five percent of the world’s autistic children are thought to reside in low- and middle-income countries (LMIC) (4). Despite growing global efforts to enhance early detection, diagnosis, and treatment of autism, the majority of research is disseminated from high-income countries. This leaves LMICs disproportionately underrepresented in research with downstream consequences including slowed innovation and progress in clinical care and policy. Several factors contribute to the under-identification of autism in LMICs including limited neurodevelopmental workforce and expertise, sociocultural barriers and disability stigma, and lack of culturally appropriate diagnostic tools (3, 5–11). To address these challenges, it is essential to build capacity among general care providers and other non-specialists to detect and diagnose children at increased likelihood of autism. Culturally appropriate, feasible open-source assessment tools are fundamental to advancing this goal in LMICs.

Best-practice autism diagnosis is a comprehensive process that combines caregiver interviews and observational assessment, often involving use of standardized diagnostic tools, with expert clinical judgment (12, 13). The Autism Diagnostic Interview-Revised (ADI-R) and the Autism Diagnostic Observation Schedule, Second Edition (ADOS-2) are two gold-standard autism assessment tools used to inform clinical decision-making (13, 14). However, these tools are expensive, time-intensive, require substantial training and clinical expertise, and are not validated for use in many languages and cultures. In resource-constrained settings, the shortage of trained professionals and the extensive time required for both training and administering these diagnostic tools pose significant challenges. The high cost of copyrighted assessment tools (i.e., some exceeding four times the annual per capita healthcare expenditure for children in many LMICs), additional per-use fees, and charges for adapting or translating these tools limit their clinical utility in resource-constrained settings. Furthermore, these widely used autism assessment tools have been developed and validated on predominantly white English-speaking populations, limiting appropriateness and potential accuracy across LMIC settings where diverse cultural, social, biological, and environmental factors may influence the presentation of autism (8, 11, 15) and utility of clinical tools and diagnostic approaches. These barriers underscore the need for brief, low-cost, open-access assessment tools that can be administered by non-specialists and flexibly adapted for diverse contexts.

Despite the clear need for feasible and culturally grounded autism assessment tools, cultural adaptation of tools has not been widely undertaken because it is methodologically complex, resource-intensive, and often constrained by copyright restrictions and limited local research capacity. Rigorous adaptation requires iterative piloting, stakeholder engagement, and psychometric validation (16–18). Cultural adaptation of assessment tools goes beyond language translation; it involves integrating cultural values, customs, traditions, beliefs, and social norms to ensure context-grounded validity (9, 18). The translation with cultural adaptation approach (TCA) addresses these constructs by rigorously examining not only linguistic equivalence but also how cultural understanding and perspectives may influence how the instrument performs in a new context (18). TCA generally includes translation by multiple translators with diverse backgrounds, consensus meetings, pre-testing with individuals from the target population, and subsequent empirical evaluation of the revised instrument’s psychometric properties. Additionally, in the limited studies that have culturally adapted standardized autism assessment tools in LMICs (19), the adaptation process was further strengthened by engaging caregivers to provide information on cultural perspectives related to activities and materials used in assessment tools.

Like many global resource-constrained settings, Kenya, an LMIC in East Africa, faces significant challenges in autism detection and diagnosis (20–22). While the awareness of autism and other neurodevelopmental disabilities is growing, access to autism services and care, including diagnostic evaluations, remains limited. This challenge is, in part, due to both the paucity of trained neurodevelopmental professionals and standard diagnostic tools validated for this setting (7, 23, 24). To date, there have been no known studies conducted to adapt or validate observational autism assessment tools in Kenya (25). The objective of this study was to linguistically and culturally adapt the ASD-PEDS (26), an open-source autism observational assessment tool, for use in western Kenya. Specifically, we conducted 1) a translation of the ASD-PEDS into KiSwahili, 2) cognitive interviews with key stakeholders to understand culturally normative social communication and play behaviors of young children and to identify key aspects of the ASD-PEDS requiring adaptation, and 2) a field test of the ASD-PEDS with young children with and without autism in western Kenya.

## Materials and methods

### Setting

This study was conducted within the Academic Model Providing Access to Healthcare (AMPATH) program, a long-standing partnership of over thirty years between Moi Teaching and Referral Hospital (MTRH), Moi University School of Medicine, and a consortium of institutions in North America, led by Indiana University. MTRH, AMPATH’s flagship partnership, serves a catchment area of 25 million people across western Kenya, eastern Uganda, northern Tanzania, the Democratic Republic of Congo and southern Sudan (27–29). Within AMPATH, a team of diverse interdisciplinary pediatric professionals from the U.S. and Kenya have collaborated on child disability research for nearly a decade. The research team in the present study includes expertise in autism diagnosis and autism service delivery systems (RMK, EO), pediatric neurodevelopmental disabilities (KFL, EO, MSM, AN) and child psychiatry (CS).

### Study design

**Figure 1:**
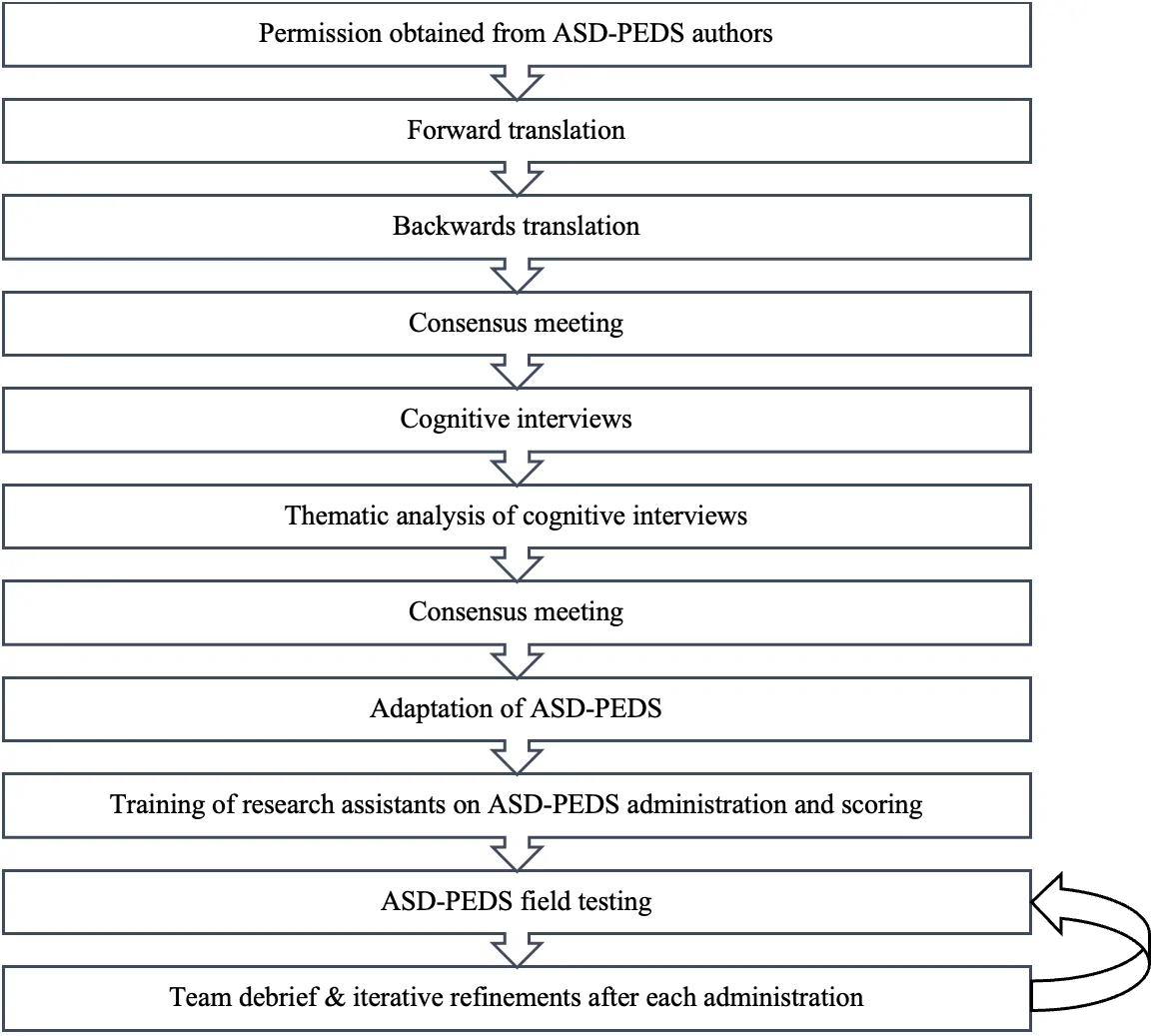
Process diagram of ASD-PEDS Swahili translation and cultural adaptation.

This mixed-methods study was approved by the Indiana University Institutional Review Board (#20917) and Moi University Institutional Research and Ethics Committee (IREC 664/223). Permission to translate and adapt the ASD-PEDS was provided by its original authors. We followed a multistep method of cultural adaptation for standardized assessment tools that have been successfully utilized by our team in similar studies (30, 31). Specifically, the ASD-PEDS was iteratively adapted through language translation, cognitive interviews with Kenyan caregivers and clinicians, and field testing with Kenyan children, which was augmented by continuous intensive expert review and consensus meetings, as shown in **Figure 1**.

### Measures

#### ASD-PEDS

The ASD-PEDS is an open-source, semi-structured observational tool designed for trained clinicians to assess autism-related behaviors in toddlers and young children. While the tool was developed for children 14–36 months of age, it has shown promise for use with minimally verbal young children older than 36 months (26). The ASD-PEDS includes nine standard play-based activities, administered over 15–30 minutes, intended to elicit social communication and interaction between the child, their caregiver, and the examiner as well as assess for the presence of restricted and repetitive behaviors. ASD-PEDS activities include free play, calling name, directing attention, joint play, familiar play routine, ready-set-go routine, requesting, ignoring, and an optional caregiver play activity. Low-cost, commonly available toys are used to create the assessment kit from a list of suggested materials in three categories: play materials, requesting materials, and ready-set-go materials. Rather than prescribing exact materials to be used, the ASD-PEDS manual provides suggested materials (i.e., toys and activities) according to their functional properties. This function-based approach provides flexibility, allowing examiners to select locally appropriate materials that aligned with the ASD-PEDS suggested material. In the present study, the original ASD-PEDS kits were sourced predominately from toys and materials found in the US, though some local materials were added or swapped throughout the adaptation process.

Seven key behaviors are rated during the assessment: (1) socially directed speech and sounds, (2) frequent and flexible eye contact, (3) use of gestures integrated with eye contact and speech/vocalization, (4) unusual vocalizations, (5) unusual or repetitive play, (6) unusual or repetitive body movements, and (7) unusual sensory exploration. For each behavior, a score from 1 to 3 is assigned (1 = behavior not present, 2 = behavior present but at a subclinical level, 3 = behavior clearly consistent with autism). A total score ≥ 13 is considered to indicate increased likelihood of autism. The ASD-PEDS is designed to be used as one component of an autism diagnostic evaluation where assignment of diagnosis is made based upon the clinician’s overall clinical judgment made via synthesis of findings across multiple sources of data [i.e., medical and developmental history, current DSM-5 autism symptoms ascertained via diagnostic interview, and observational assessment results] (26, 32).

#### 2015 Poverty probability index

The Poverty Probability Index (PPI) is a contextually sensitive tool individualized for a country to predict the likelihood of a household living under the poverty line (33, 34). It is composed of 10 easy-to-answer questions that can be administered in under 10 minutes. PPI scores range from 0–100 with higher scores indicating lower probability that a household is below the poverty line. In the research setting, the PPI estimates each participants’ level of household consumption as a proxy for economic status and provides likelihood of participant being qualified under each quintile of population consumption distribution (i.e., ranging from the lowest, or poorest 20%, to the highest, or richest 20%). It is important to note that PPI scores are not averaged because the scores are ordinal and converted into poverty likelihoods, which are not evenly distributed across score ranges (33, 35).

### Phase 1: ASD-PEDS translation and adaptation

#### Forward and back translation

Translation procedures were informed by established guidelines for cross-cultural adaptation of assessment instruments and emphasized functional rather than literal equivalence between the English and Kiswahili versions (36). The ASD-PEDS administration guidelines were independently translated from English into Kiswahili by two bilingual Kenyan research assistants with extensive experience in translation and research implementation. The two forward translations were compared and reconciled through consensus discussion to create a single preliminary Kiswahili version. This version was then independently back-translated into English by a trained Kenyan translator who was blinded to the original English version. The original English version and back-translated version were systematically compared by members of the U.S.-Kenyan research team to identify discrepancies, ambiguities, and ensure culturally sensitive language. Translation discrepancies were documented and discussed during consensus meetings, and modifications were made when necessary to improve conceptual and functional equivalence while preserving the intent of the original administration guidelines (36).

Multiple procedures were used to evaluate the validity and appropriateness of the translated administration guidelines. During field testing, participant feedback was used to identify wording requiring clarification and to evaluate the cultural appropriateness of assessment tasks and materials. Additional validation occurred through assessor documentation of implementation challenges and language-related concerns. Findings were reviewed during iterative study meetings, resulting in additional refinements to the translated administration materials, including development of a bilingual English-Kiswahili.

#### Cognitive interviews

Cognitive interviewing involves asking participants to think aloud as they answer questions in order to ascertain how participants understand, interpret, and respond to specific questions or tasks (30, 37). Cognitive interviews identify recall and thought processes related to an item and can uncover potential response errors. A cognitive interview guide was developed (and translated) by the study team to inform ASD-PEDS adaptation needs. Interview questions assessed culturally normative play and social behavior in Kenyan children and cultural familiarity and appropriateness of the ASD-PEDS instructions, tasks, and materials. Two Kenyan research assistants with prior experience in qualitative interviewing were trained to administer the cognitive interviews through a series of virtual workshops on obtaining consent for interviews, core concepts of cognitive interviewing, administration of the interview guide, and strategies to address common interview challenges. The interview guide was piloted by the research assistants and reviewed by the research team to refine interview questions and techniques.

Cognitive interview participants were instructed to “think aloud” during the interview. A practice activity was performed to facilitate participants’ understanding and comfort with the think-aloud method. Once comfortable, the participants proceeded with the cognitive interview questions while describing their thought process audibly to capture perspectives on ASD-PEDS and to provide real-time insight into their reasoning (37, 38). Participant responses were further probed when the interviewer judged that further elaboration was needed to fully ascertain detailed responses (37). Interviews were audio-recorded, transcribed, and translated from Swahili to English for analysis.

#### Participants

Ten participants were recruited to participate in cognitive interviews from partner MTRH clinics and our team’s autism research studies; see **Table 1** for participant demographics. Caregiver participants (N = 5; 80% female; mean age=40.2 years, SD = 2.0) included those with (n=3) and without (n=2) children diagnosed with autism or another known neurodevelopmental disability. Five clinicians (mean age in years=41.6, SD = 11.1; years in practice: 10.8, SD = 5.0), employed at MTRH that serve children with disabilities included a psychology nurse, occupational therapist, child life specialist, psychiatrist, and mental health clinical officer. All clinicians (N = 5, 100%) provide regular care to children in the study age range (i.e., 14–72 months) with 60% (n=3) and 80% (n=4) receiving previous training in child development and autism, respectively. Participants were compensated 1000 Kenyan shillings (about $8 reflecting standard accepted compensation rate for the setting) for participating in the cognitive interview.

**Table 1:** Baseline demographics of cognitive interview participants, N = 10.

Demographics	Caregivers (n=5)	Clinicians (n=5)
Age, mean (SD)	40.2 (2.0)	41.6 (11.1)
Sex, n (%)		
Male	1 (20)	2 (40)
Female	4 (80)	3 (60)
Primary language, n (%)		
KiSwahili	5 (100)	2 (40)
Luhya	0 (0)	1 (20)
English	0 (0)	3 (60)
Marital status, n (%)		
Married	4 (80)	n/a
Not married	1 (20)	n/a
Employed, n (%)	4 (80)	5 (100)
Number of children, mean (SD)	3.2 (1.3)	n/a
Years in practice, mean (SD)	n/a	10.8 (5.0)

#### Analysis & adaptation

Cognitive interview transcripts were independently analyzed by two members of the research team using Braun and Clarke’s six-step thematic analysis framework (39). Two members of the research team independently reviewed all transcripts to develop familiarity with the data and identify key quotes relevant to the clarity, cultural appropriateness, and usability of the ASD-PEDS (Step 1). The same two coders then conducted inductive open coding independently, generating initial codes directly from participant responses rather than applying a predefined coding framework (Step 2). Following independent coding, coders met to compare coding decisions, discuss discrepancies, and reach consensus through discussion and review of the original transcripts. Formal inter-coder reliability statistics were not calculated, as the analysis prioritized consensus-based thematic development. Codes were subsequently organized into preliminary themes (Step 3), which were reviewed and refined collaboratively by U.S. and Kenyan research team members to ensure cultural relevance and accurate interpretation of findings (Steps 4–5). Formal member checking with interview participants was not conducted; however, findings were reviewed by Kenyan team members with expertise in the local context to support interpretation and confirm the relevance of identified themes. Although data collection and analysis were conducted iteratively, formal assessment of thematic saturation was not conducted because the primary purpose of the cognitive interviews was to identify opportunities for ASD-PEDS adaptation rather than to develop a comprehensive theory of participant experiences. Nonetheless, later interviews yielded few novel adaptation recommendations. A final thematic report was produced (Step 6), and themes were systematically mapped to adaptation activities (39). Specifically, participant feedback regarding item wording, examples of developmental expectations and milestones, cultural relevance, and administration procedures informed revisions to the ASD-PEDS prior to field testing. All proposed adaptations were reviewed and approved by the study team before implementation.

### Phase 2: ASD-PEDS field testing

After the cognitive interviews, field testing using the translated and adapted ASD-PEDS tool was conducted to identify any additional areas requiring adaptation. Two research assistants were trained in administration and scoring of the ASD-PEDS by a licensed clinical psychologist with decades of expertise in early autism assessment and diagnosis (RMK). Training involved didactic education, case-based review and scoring of video-recorded ASD-PEDS assessments and supervised live administration and scoring practice with feedback. Sixty percent (n=12) of administrations were completed under live supervision and expert consensus co-scoring. Caregivers completed a brief survey on child and family demographics (including the PPI) and child medical diagnosis. The ASD-PEDS was administered to children with the caregiver present. Following the assessment, caregivers were informally asked about their experience participating in the assessment to identify any aspects of the administration and materials that warranted further consideration for adaptation. Team debriefing occurred after each ASD-PEDS administration, and additional refinements were made.

#### Participants

Twenty children, 14–72 months of age (mean=46 months, SD = 16), with and without a prior diagnosis of autism were recruited from MTRH pediatric neurology, psychiatry, and occupational therapy clinics as well as through our team’s child development studies; see **Table 2**. Thirteen children (65%) had an existing medical diagnosis of autism determined by a pediatric sub-specialist; ten of those children also had co-occurring conditions including developmental delay, language delay, and epilepsy. In addition to their primary language, all households reported speaking at least one other language, including Kiswahili, English, Kalenjin (unspecified dialect, Marakwet, and Keiyo), Kisii, Luhya, and Kikuyu. Participants were compensated 1000 Kenyan shilling (about $8) for participating in field testing.

**Table 2:** Participant demographics for ASD-PEDS field testing (N = 20).

Demographics	n=20
Childs age (in months), mean (SD)	46.1 (15.9)
Caregivers age (in years), mean (SD)	38.2 (10.9)
Childs sex, n (%)	
Male	11(55)
Female	9 (45)
Child diagnosis, n (%)	
Autism	13 (65)
Non-autism	7 (35)
Primary language, n (%)	
Kiswahili	17 (85)
English	1 (5)
Other	2 (10)
Poverty probability index (n=18) ^1^	
Quintiles for population consumption distribution across individuals, % ^2^	
0-20% (bottom 20% - poorest)	4.4
20-40% (2nd Quintile)	10.6
40-60% (Average)	22.3
60-80% (4th quintile)	33.1
80-100% (Top 20% - Richest)	29.7
Highest educational level of the female household head/spouse, n (%)	
Primary	1(0.6)
Secondary, post-primary or vocational	8 (0.4)
College level or higher	9 (0.5)
Highest educational level of any member of the household, n (%)	
Primary	1 (0.6)
Secondary, post-primary or vocational	8 (0.44)
College level or higher	9 (0.5)
Over the past 7 days, the household either purchased/consumed/acquired, n (%):	
Bread	17 (94)
Meat or fish	12 (66)
Ripe bananas	13 (72)
Household owns, n (%)	
Towel	16 (89)
Thermos flask	6 (33)
Predominant wall material of the main dwelling unit, n (%)	
Finished walls (cement, stone with lime/cement, bricks, cement blocks, covered adobe, or wood planks/shingles)	13 (72)
Uncovered adobe, plywood, cardboard, reused wood, or corrugated iron sheets	4 (22)
Natural walls (cane/palm/trunks, grass/reeds, or mud/cow dung), no walls, bamboo with mud, stone with mud, or other	1 (5)
Predominant floor material of the main dwelling unit, n (%)	
Natural floor (earth/sand or dung) or palm/bamboo	1(6)
Other (including wood planks/shingles, parquet or polished wood, vinyl or asphalt strips, ceramic tiles, cement, or carpet)	17 (94)

2 Two participants did not complete the PPI; therefore, results are based on 18 respondents.

2 The PPI generates probability estimates for each consumption quintile rather than classifying participants into one definitive quintile. Because these are modeled likelihoods derived from ordinal PPI scores and not counts of individuals, quintile-specific n values cannot be reported.

#### Analysis & refinement

Descriptive statistics were calculated for demographic variables, ASD-PEDS items and total scores, and ASD-PEDS categorical autism outcome (autism, non-autism). Agreement between ASD-PEDS classification (i.e., total score 7-12 = non-autism; 13-21 = autism) and child’s existing medical diagnosis was calculated. Continuous variables were summarized using means, standard deviations, and 95% confidence interval (CI), while categorical variables were reported as frequencies and percentages.

## Results

### Phase 1: ASD-PEDS adaptation

#### Cognitive interviews

Analysis of cognitive interviews revealed five main themes: (1) early social communication skills in Kenyan children, (2) traditional play behaviors and social routines of Kenyan children, (3) ASD-PEDS materials, (4) ASD-PEDS activities, and (5) ASD-PEDS administration. **Table 3** presents themes and representative quotes from cognitive interviews.

**Table 3:** Themes and representative quotes from cognitive interviews.

Theme	Representative quotes
Early social communication skills in Kenyan children	
Gestures	“He/she may point, they can stretch and pick it or may make some sound while pointing whatever he/she wants. “ -Caregiver, 42-year-old female, caregiver to child with typical development “Point, the gesture ‘Come’, the gesture ‘bye’, they shake their head and nod to mean yes and no. they look away if they don’t want something. They shrug their shoulders to express dissatisfaction.” -Clinician, 38-year-old occupational therapist
Eye contact	“…in the African traditions we feel like when you maintain eye contact you are rude or you are trying to slice authority, but when they are talking to their mother, like my children look me in my eye.” -Clinician, 33-year-old senior medical officer in pediatric psychiatry “Those who are disciplined don’t look into the eye directly. They can’t. They can’t. But every parent has their own rules and regulations that govern a home.” -Caregiver, 38-year-old female; caregiver to child with neurodevelopmental diagnosis
Traditional play behaviors and social routines in Kenyan children	
Play	“When they are together, they play things like kalongoo, playing with the ball, things like those ones” Caregiver, female, 38 years old; caregiver to child with typical development “One, they like playing in mud, making toys by molding with mud. They like making those toys together imitating each other. They like competing and running and also playing football.” -Caregiver, 42-year-old female; caregiver to child with typical development “They like playing with toys. If they are boys, they like moving around with the car toys or making their own from wires. Girls like carrying dolls and then together they like imitating a family like making daddy, mum, siblings.” -Clinician, 57-year-old psychological nurse
Social routines	“You may find yourself hiding somewhere and say ‘are you ready? Are you ready? Do I come?’ then that child will be looking for you from the corners so that they can find you. Then you hear ‘taboo!’ such things.” Caregiver, female, 38 years old “Hide and seek is there. Peekaboo is new in our country. You know it has not been there. It is something which is coming up, but I have seen the young parents do it especially mother and child.” -Clinician, 57-year-old psychological nurse
ASD-PEDS materials	
Toys	“It depends on where one is coming from. If they are coming from within the town and they can afford those resources. Like where I come from, one cannot access these things. One who can afford is not the same as one who cannot afford. There are people who can afford toy cars for two thousand shillings but there are those who cannot afford that one. Alternatively, if they find a cheaper one, they will look for that one. It depends on ones’ background” -Caregiver, female, 42-year-old, caregiver to child with neurodevelopmental diagnosis “child will make a ball for themselves and play on soil” -Caregiver, female, 42-year-old; caregiver to child with neurodevelopmental diagnosis “[ready-set-go toys are] not readily available” -Clinician, 38-year-old occupational therapist
Snacks	“sponge cakes, biscuits“ -Caregiver, 38-year-old female, caregiver to child with neurodevelopmental diagnosis
ASD-PEDS activities	
Free-play	“all the toys are there, and he/she will choose which one pleases him/her first.” -Caregiver, female, 38 years old “the child will be playing with it because that is the nature of children.” -Clinician, 31-year-old mental health clinical offices
Name calling	“Because every child or every human being who has a normal mind will obviously respond to their name and then whether you are related, familiar or what” -Clinician, 57-year-old psychological nurse
Directing attention	“A child of around six years will try to see what the doctor is pointing.” -Clinician, 31-year-old mental health clinical officer
Joint play/turn taking	“The normal children will want but those children with autism may not even bother about that.” -Caregiver, female 42-year-old, caregiver to child with neurodevelopmental diagnosis
Familiar play routine	“[This activity is] assessing for the social skill and interest in activities of a child” -Clinician, 57-year-old psychological nurse
Ready-set-go routine	“This doctor is using that activity to initiate a play and see if the child can sustain and maintain the same play” -Clinician, 57-year-old psychological nurse
Requesting	“Food is usually attractive to anyone and especially that container is to make an attraction. So, when the doctor puts it there and it has a lid, the doctor is looking for communication and skills about a child.” -Clinician, 57-year-old psychological nurse
ASD-PEDS administration	
Child & clinician interaction	“There are other children who will respond to one name and not respond to another. I would be careful to ascertain which name the child answers before the clinician starts calling because a lot of times children identify with their nickname as opposed to their names.” -Clinician, 38-year-old occupational therapist “[During familiar play routine],it is nice if a doctor asks the caregiver which game is familiar to the child and then they can play it. That brings familiarity to the child. Children are so used to routines and them like familiarity. …if the caregiver started playing peekaboo and then the doctor comes in as the parent and child are already playing, that would yield some good results.” - Clinician, 49-year-old social worker and child life specialist
Clinical setting	“What level of privacy would it be because if you are letting a child be within a room, what space constrains are we talking about? … If they are on the floor, what sort of mat would you have?….How about the noise levels in the room? We are talking about children on the spectrum. What noise proofing has been done in the room, how many clinicians would be in the room at the same time?” -Clinician, 38-year-old occupational therapist

#### Early social communication skills in Kenyan children

Gestures such as pointing and nodding and as well as overtures such as giving and showing were described to be culturally normative social communication behaviors. Participants described that modulation of eye contact in young children varies based on age, experiences, and differences in cultural expectations. For example, children may demonstrate reduced eye contact with authority figures, including doctors, due to learned expectations, lack of exposure to healthcare professionals, or previous unfavorable experience with medical treatment.

#### Traditional play behaviors and social routines in Kenyan children

Kenyan children were reported to play by making toys (e.g., balls and dolls), engaging in outdoor games, playing in soil, and doing chores. Typical imaginative play includes pretending to be parents, teachers, or doctors. Social routines reported between parents and children included games such as hide and seek, tickling, and chasing. Peek-a-boo was described as a newly emerging social game most commonly played by younger families.

#### ASD-PEDS materials

Participants suggested ASD-PEDS materials such as balls, plastic containers, and some ready-set-go toys were familiar to most children. Toys such as a glitter wand, blocks, and toys that launch (e.g., foam rocket) were not considered widely available or familiar. Participants described access to such toys contingent on a family’s ability to afford them or a child’s geographic context. Caregivers and clinicians reported on the use of culturally relevant and widely available snacks such as biscuits and crisps.

#### ASD-PEDS activities

Participants reported that the ASD-PEDS activities were described to elicit the expected social and play behaviors of young Kenyan children, with no suggestions for adaptation.

#### ASD-PEDS administration

Participants identified that a child’s familiarity with the adult administering the test could influence the presentation of the child’s behavior. Participants noted that children might be reluctant to take food from unfamiliar adults, suggesting that giving the food container to the caregiver, who then offers it to the child could improve participation in requesting. Participants were also concerned that children may not easily engage in familiar play routines with an unfamiliar adult. Additionally, participants suggested that children are likely to respond to a familiar person (i.e., caregiver versus clinician) and might be more responsive to a commonly used nickname rather than their formal name. Specific concerns identified about the clinical setting during administration included distractions within the exam room (i.e., noise level, the number of clinicians in the shared exam space, wall hangings, phones, and computers). Concerns were raised regarding the location of play activities, particularly with shared exam space and floor play (i.e., both common in Kenyan clinical settings). To support directing attention, it was recommended to place a colorful, bright, interesting poster or large object in the room.

#### Adaptation and refinement

A private exam room was identified to administer the testing privately. A playmat was added to the ASD-PEDS kit. Before beginning the assessment, an engaging item was selected to facilitate directing attention. A bright poster was added to the kit to use if an engaging object was not available in the selected testing space. Test administrators ensured they used the child’s preferred name. Culturally appropriate snacks were used as part of the assessment process. Initially, a balloon was used as a ready-set-go toy due to inconsistent familiarity with other suggested ready-set-go materials. A culturally familiar play routine (i.e., hide-and-seek) was trialed.

### Phase 2: ASD-PEDS field testing and iterative refinement

#### Field testing

ASD-PEDS classification (i.e., autism; non-autism) matched children’s known medical diagnosis in 100% of cases. **Table 4** displays ASD-PEDS scores and classification by diagnostic group.

**Table 4:** ASD-PEDS field testing results (N = 20).

	Autism (n=13)			Non-autism (n=7)		
ASD-PEDS autism classification, n (%)	13 (100)			0 (0)		
Item	Mean	SD	95% CI	Mean	SD	95% CI
ASD-PEDS total score	18.00	2.65	17.72, 18.74	8.00	1.15	7.69, 8.31
ASD-PEDS item scores						
Socially directed speech and sounds	2.69	0.48	2.60- 2.78	1.43	0.8	1.22, 1.64
Frequent and flexible eye contact	2.54	0.66	2.41- 2.67	1.00	0.00	—
Use of gestures & integration with eye contact and speech /vocalization	2.54	0.52	2.44, 2.64	1.00	0.00	—
Unusual vocalizations	2.61	0.51	2.52, 2.71	1.00	0.00	—
Unusual or repetitive play	2.46	0.51	2.36, 2.56	1.43	0.51	1.28, 1.57
Unusual or repetitive body movements	2.46	0.52	2.36, 2.56	1.00	0.00	—
Unusual sensory exploration or reaction	2.77	0.44	2.68, 2.85	1.14	0.44	1.04, 1.25

#### Adaptation and refinement

**Figure 2:**
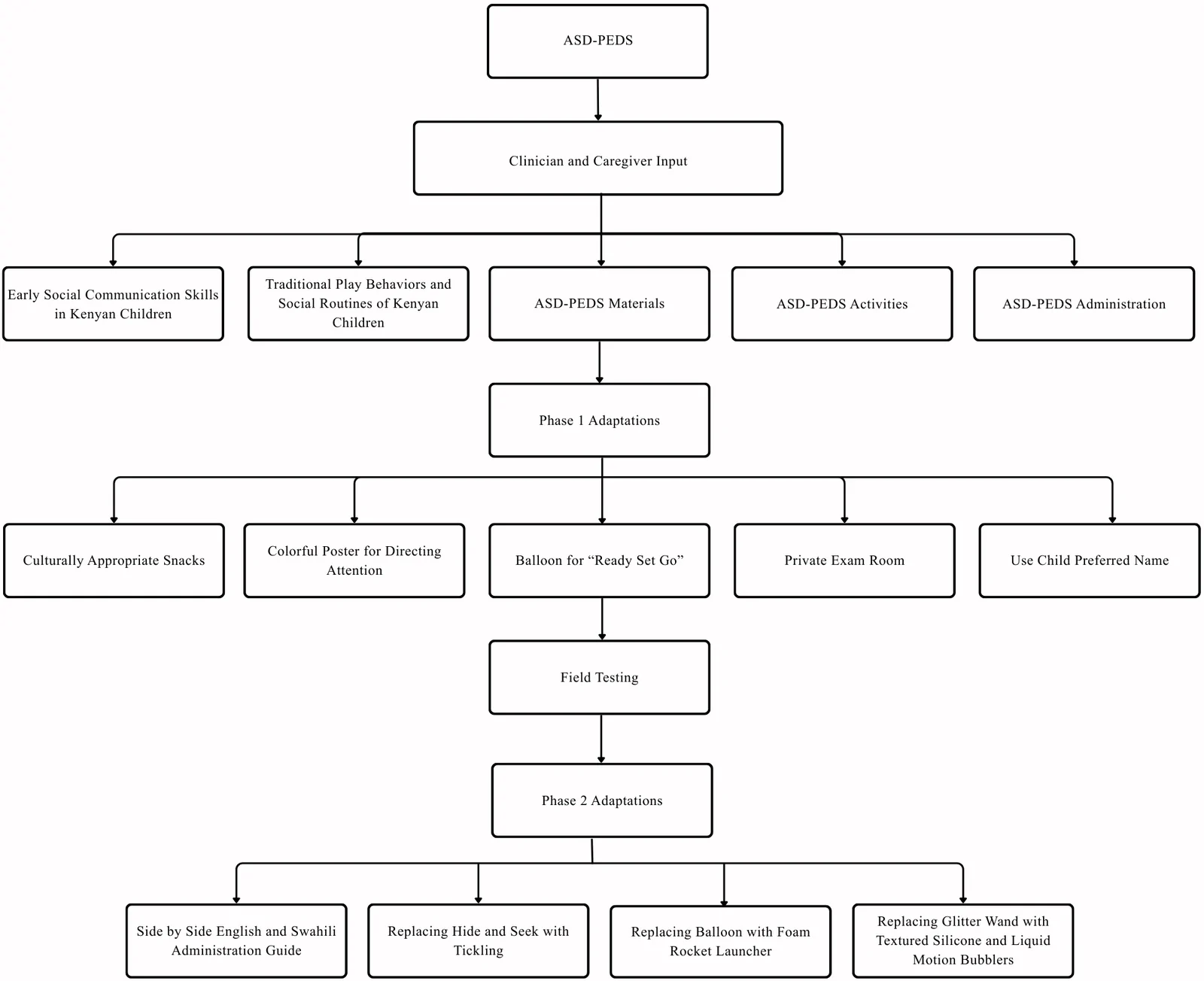
ASD-PEDS adaptation process and results.

During field testing, further refinements in translation were made. The ASD-PEDS administration form was revised to feature side-by-side English and Swahili translations, based on feedback from clinicians who reported regularly switching between the two languages during assessments depending on the preferred language of the child and caregiver. Hide and seek was initially trialed as the familiar play routine activity; due to limitations in the size of the room, the team found that a tickling routine worked better in the space and that children and caregivers easily recognized this routine. A balloon was initially trialed for “ready-set-go” routine during phase one but was discontinued due to concerns for hygiene and safety. Despite unfamiliarity reported with the foam rocket launchers, when trialed, these functioned effectively and as expected, and their novelty was not judged to impact the assessment. A glitter wand was initially included in the ASD-PEDS kit, however there were concerns regarding safety (i.e., concerns the wand may break or injure administrators if hit by the wand); glitter wands were replaced with locally available toys (i.e., textured silicone with liquid motion bubbles). The complete process and adaptation results are outlined in **Figure 2**.

## Discussion

The objective of the present study was to linguistically and culturally adapt the ASD-PEDS, an open-source autism observational assessment tool for use in western Kenya (26). To our knowledge, this is the first study to adapt an open-source autism assessment tool for young children in a global resource-constrained setting. The adaptation process involved forward and backward translation, cognitive interviews with caregivers and clinicians, and field testing in children with and without autism. Findings suggest that early social communication and traditional play behaviors observed in Kenyan children can be assessed using the ASD-PEDS with minimal modifications required for cultural relevance, demonstrating preliminary evidence of feasibility. Minor adaptations to the ASD-PEDS materials were necessary to reflect local norms and improve accessibility. Cultural expectations regarding child interactions with authority figures, such as clinicians, were considered as part of the administration process. Additionally, bilingual administration instructions in Swahili and English were incorporated to accommodate clinicians who routinely switch between languages during assessments. Environmental modifications, including the use of private exam rooms and distraction-reducing strategies, were implemented to support optimal assessment conditions.

## Translation

Existing cultural adaptation studies emphasize that translating assessment tools into regionally relevant languages can enhance accessibility and promote widespread use, with additional translations into local languages further increasing reach (40). Rigorous translation procedures typically involve multiple culturally competent bilingual translators, followed by expert review and pilot testing to ensure cultural appropriateness and minimize bias (41). Many studies also prioritize functional equivalence, maintaining the intended meaning and use of each item while preserving alignment between the source language and translated text (42, 43). Additionally, previous work translating neurobehavioral assessments in Kenya has noted the intentional retention of certain English words—given that English is the primary language of instruction and that some terms are more familiar in English than in local languages (42).

Guided by these established standards and broader methodological approaches to adaptation of tools in the literature, the ASD-PEDS was first translated into Swahili using a rigorous, multi-step process emphasizing functional equivalence and alignment between English and Swahili versions. To further strengthen the translation and ensure cultural and clinical relevance, we incorporated extensive stakeholder engagement throughout the adaptation process. This included cognitive interviews, iterative refinement during field testing, and consensus discussions across our US–Kenyan research team. Although our translation process did not involve intentional retention of English terms, assessors frequently code-switched between English and Swahili in practice. These observations directly informed the development of a bilingual administration guide presenting both languages side by side, supporting common linguistic norms in Kenyan clinical settings. Although this initial translation is an essential first step to adaptation of the ASD-PEDS, English and Swahili represent only two of the more than 60 languages spoken in Kenya (10), indicating that broader accessibility and scalability will require future translation into additional regional languages in the future.

### Cognitive interviews

Cognitive interviews have been widely incorporated into Kenyan cultural adaptation studies, including work on social support measures, neurobehavioral assessments, and other clinical tools (30, 44, 45). This methodology provides an essential strategy for identifying cultural nuances that may not surface through translation alone. As shown in both previous studies and the present work, qualitative engagement with key stakeholders enhances adaptation by revealing context-specific considerations that inform item interpretation and relevance (42, 44, 45).

Our findings can be situated within cross-cultural developmental that propose that core developmental processes are largely universal, while the manner in which developmental behaviors are expressed, encouraged, and interpreted is influenced by cultural context (16). Within this framework, foundational social communication behaviors that emerge early in development, including joint attention, gestural communication, social reciprocity, and shared affect, are expected across cultures, although specific behavioral manifestations may vary according to local norms and expectations (46–48). Consistent with this perspective, caregivers and clinicians in our study described social communication behaviors that closely aligned with those assessed by the ASD-PEDS, supporting the cross-cultural relevance of the underlying developmental constructs measured by the tool.

Cognitive interviews suggested that early social communication behaviors in Kenyan children, including use of eye gaze, directed facial expressions and shared affect, joint attention, gestures (e.g., pointing) and showing, are culturally normative and well-aligned with behaviors assessed by the ASD-PEDS (26, 49, 50). These early emerging social communication behaviors are also found in the Developmental Milestones Checklist, a developmental screening tool specifically developed and validated for use in Kenya, further supporting the appropriateness of the ASD-PEDS activities in this context (26, 49). Notably, our cognitive interview participants described that Kenyan children’s eye contact varies by age, experience, and cultural norms, and may be limited during interactions with clinicians due to perceived authority and expectation to demonstrate respect, unfamiliarity with healthcare providers, or prior negative experiences in the medical setting. These findings align with other studies of social communication in Asian children where variations in modulation of eye contact was described to reflect subtle cultural norms rather than social communication differences or impairment (51).

Cognitive interviews conducted in the present study further identified the need for careful consideration of contextual relevance of the ASD-PEDS activities and materials. For example, when asked about normative social routines for young children, respondents described that hide-and-seek is a common routine among most families; yet peek-a-boo routines are predominantly played by younger caregivers and thus may not generalize across families. Original ASD-PEDS materials, presented in cognitive interviews, were sourced from the US. As expected, participants identified that some of these materials were unfamiliar, including the foam rocket launcher toy. Participants also recommended use of culturally appropriate snacks.

These findings are consistent with prior cultural adaptations of autism assessment tools, including the adaptation of the ADOS for South Africa (19). In that study, several standard ADOS activities were considered unfamiliar or perceived as difficult to administer. For example, tooth-brushing routines varied depending on access to a sink and the methods used, and birthday party enactments were considered challenging because few families reported regular participation in these activities. Cultural differences also influenced children’s responses to testing materials: action figures elicited concern among caregivers due to associations with violence; toys such as frogs were avoided because they were viewed as frightening or inappropriate; and strong gender norms shaped expectations about which toys were suitable for boys versus girls. Unlike the ADOS, the strength of the ASD-PEDS includes explicit flexibility to select activities or items depending on familiarity to children and caregivers and available in the setting used. However, while modifications to ASD-PEDS activities were minor in the present study, it is possible that construct validity could have been impacted; as such, this should be a target of future study in a larger psychometric validation.

### Field testing

Our goal in the present study was to conduct a field test of the ASD-PEDS to provide further targets for adaptation and refinement. During field testing of 20 participants, the ASD-PEDS categorical outcome matched child’s previous clinical autism diagnosis (i.e., autism present/absent) in all cases. Aligned with our cognitive interview results, field testing further confirmed that normative social communication behaviors such as gestures, pointing, responding to one’s name, and joining others in play were demonstrated consistently by children without a clinical diagnosis of autism. Despite cognitive interviews elucidating potential concerns regarding culturally normative variations eye contact, all of the non-autistic children were rated as having “flexible and frequent eye contact” suggesting that these potential subtle variations did not impact rating of this behavior in our sample of young children in western Kenya. Similarly, restricted and repetitive behaviors were rarely observed and scored in this group. Across all 13 autistic children who participated in field testing, behaviors commonly associated with autism and measured by the ASD-PEDS, including inconsistent integration of verbal and nonverbal social communication behaviors, use of atypical vocalizations, presence of repetitive behaviors, and atypical sensory exploration or responses, were consistently observed. Together, the field-testing findings provide promising initial evidence for the utility of the culturally adapted ASD-PEDS for identifying autism in young children in western Kenya; however, further pilot testing will be essential for evaluating the tool’s suitability within a targeted cultural context and determining readiness for broader implementation (10).

### Limitations & setting-specific challenges

Although our findings are promising, several setting-specific challenges and methodological limitations are noteworthy. Overall, the majority of ASD-PEDS adaptations were feasible to implement within the Kenya context. However, limited access to a private examination room, which supports a distraction-free clinical environment, may be a significant challenge for scalability given that shared exam rooms are common in these settings. Future work must address how diagnostic assessment tools can be used as part of autism evaluations when physical space limitations may impact utility and validity of such practices.

Our sample included relatively well-resourced families, including those who participated in prior autism research studies and who had the means to travel to and attend study visits conducted at a national hospital. Over 85% of children in our field-testing sample were from families with average or above socioeconomic backgrounds and less than 5% fell within the lowest resourced quintile, as measured by the PPI. In contrast, 20% of Kenya’s national population and 20-30% of households in the counties where our participants reside fall within this lowest quintile (34). As such, our sample does not reflect national or local socioeconomic makeup, with under-representation of participants from low-resource and remote (i.e., those living outside of urban areas, such as within villages) backgrounds. Specifically, our results should be considered preliminary due to selection bias of families with greater financial resources, flexible work schedules, and reliable transportation that allowed them to participate in research requiring in-person, time-intensive assessment. Relatedly, it is possible that our results will not generalize to those facing broader access-to-care challenges, including systemic barriers to reaching specialty diagnostic services related to cost, geographic distance, lack of transportation, and competing demands. Low resourced families are underrepresented not only in the present study but in the clinical populations from which research samples are typically drawn more broadly, limiting the generalizability of findings to the broader population of LMICs.

A second methodological limitation is that data were collected in a single clinical setting by trained research assistants rather than routine clinical providers. As research assistants may have greater protocol adherence and fewer competing clinical demands, these findings may not fully reflect feasibility or performance under real-world implementation conditions. These limitations underscore the need for expansion and replication of this work with more diverse and representative populations.

Finally, as an early-stage adaptation study, significance of diagnostic findings (i.e., agreement between ASD-PEDS classification and medical diagnosis) should be interpreted with caution and not taken as evidence of broad applicability. Diagnostic performance in this sample may have been influenced by the inclusion of children with clear autism presentations. Further, the small and non-representative clinical sample, lack of assessor blinding, reliance on pre-existing medical diagnosis without independent verification, and lack of phenotypic data to characterize child autism severity and cognitive and language functioning, are additional limitations that should be considered when interpreting findings. Similarly, while this study carefully evaluated whether neurotypical Kenyan children demonstrated typical social communication behaviors aligned with ASD-PEDS scoring guidelines, it did not explore whether autistic children in Kenya show distinct or culturally specific autism phenotypes as compared to those in other regions of the world.

## Conclusion

This study describes a rigorous process of translation and cultural adaptation of the ASD-PEDS for use in western Kenya. Engaging both clinicians and caregivers in this work allowed for adaptations that were contextually grounded, culturally responsive, and easily understood by end-users. Findings indicate that the ASD-PEDS requires only minimal adaptations to support cultural relevance and accessibility. The non-proprietary nature, brief administration time, and flexible materials and structure of the ASD-PEDS make it particularly well-suited for global and low-resource settings. Early emerging social communication behaviors measured by the ASD-PEDS were found to be culturally normative among Kenyan children. Key adaptations generated in the present study included bilingual administration guidelines, culturally appropriate snacks, adjustments to familiar play routines, and modifications to the clinical environment to reduce distractions and support engagement. This work is currently being expanded to evaluate the validity of the adapted ASD-PEDS when implemented more broadly within a hospital-based diagnostic evaluation clinic within western Kenya. Future research on use of the ASD-PEDS across global settings must explore utility across varied clinical settings and populations, including children from lower socioeconomic backgrounds and rural communities.

## Data Availability

The raw data supporting the conclusions of this article will be made available by the authors, without undue reservation.
